# The Effectiveness of Imipenem–Relebactam against Ceftazidime-Avibactam Resistant Variants of the KPC-2 β-Lactamase

**DOI:** 10.3390/antibiotics12050892

**Published:** 2023-05-11

**Authors:** Krisztina M. Papp-Wallace, Melissa D. Barnes, Magdalena A. Taracila, Christopher R. Bethel, Joseph D. Rutter, Elise T. Zeiser, Katherine Young, Robert A. Bonomo

**Affiliations:** 1 Research Service, Veterans Affairs Northeast Ohio Healthcare System, Cleveland, OH 44106, USA; 2 Department of Medicine, Case Western Reserve University, Cleveland, OH 44106, USA; 3 Department of Biochemistry, Case Western Reserve University, Cleveland, OH 44106, USA; 4 JMI Laboratories, a Subsidiary of Element Materials Technology, North Liberty, IA 52317, USA; 5 Merck & Co., Inc., Kenilworth, NJ 07033, USA; 6 GRECC, Veterans Affairs Northeast Ohio Healthcare System, Cleveland, OH 44106, USA; 7 Departments of Pharmacology, Molecular Biology and Microbiology, Proteomics and Bioinformatics, School of Medicine, Case Western Reserve University, Cleveland, OH 44106, USA; 8 CWRU-Cleveland VAMC Center for Antimicrobial Resistance and Epidemiology (Case VA CARES), Cleveland, OH 44106, USA

**Keywords:** KPC-2, carbapenemase, diazabicyclooctane, beta-lactam, beta-lactamase, relebactam, imipenem, ceftazidime, avibactam

## Abstract

Background: Ceftazidime-avibactam was approved by the FDA to treat infections caused by Enterobacterales carrying *bla*_KPC-2_. However, variants of KPC-2 with amino acid substitutions at position 179 have emerged and confer resistance to ceftazidime-avibactam. Methods: The activity of imipenem-relebactam was assessed against a panel of 19 KPC-2 D179 variants. KPC-2 and the D179N and D179Y variants were purified for biochemical analyses. Molecular models were constructed with imipenem to assess differences in kinetic profiles. Results: All strains were susceptible to imipenem–relebactam, but resistant to ceftazidime (19/19) and ceftazidime-avibactam (18/19). KPC-2 and the D179N variant hydrolyzed imipenem, but the D179N variant’s rate was much slower. The D179Y variant was unable to turnover imipenem. All three β-lactamases hydrolyzed ceftazidime at varying rates. The acylation rate of relebactam for the D179N variant was ~2.5× lower than KPC-2. Poor catalytic turnover by the D179Y variant precluded the determination of inhibitory kinetic parameters. Acyl-complexes with imipenem and ceftazidime were less prevalent with the D179N variant compared to the D179Y variant, supporting the kinetic observations that the D179Y variant was not as active as the D179N variant. Relebactam was slower to form an acyl-complex with the D179Y variant compared to avibactam. The D179Y model with imipenem revealed that the catalytic water molecule was shifted, and the carbonyl of imipenem was not within the oxyanion hole. Conversely in the D179N model, imipenem was oriented favorably for deacylation. Conclusions: Imipenem–relebactam overcame the resistance of the D179 variants, suggesting that this combination will be active against clinical isolates harboring these derivatives of KPC-2.

## 1. Introduction

The pace of β-lactamase evolution is outcompeting scientific advances in the development of novel therapeutics [1]. Serine β-lactamases that hydrolyze carbapenems are a significant threat to our antibiotic armamentarium [2,3]. KPC enzymes are the most prevalent carbapenemases in the United States and are most often found in Enterobacterales [4]. Prior to the approval of ceftazidime-avibactam, meropenem–vaborbactam, and imipenem–relebactam by the US Food and Drug Administration (FDA), clinicians had limited options to treat patients who acquired infections by a KPC-2/3 producing Enterobacterales [5]. Polymyxins, more toxic antimicrobials, were often employed. Thus, the approval of ceftazidime-avibactam, meropenem–vaborbactam, and imipenem–relebactam was a renaissance in the treatment of these infections. The β-lactamase inhibitor partners, avibactam, vaborbactam, and relebactam potently inactivated KPC-2/3, unlike the preceding β-lactamase inhibitors approved in the 1980/90s, clavulanic acid, sulbactam and tazobactam, which were hydrolyzed by KPC [6,7,8,9,10].

However, with the more frequent clinical use of these novel β-lactam-β-lactamase inhibitor combinations, there were increasing reports of ceftazidime-avibactam-resistant KPC-producing Enterobacterales isolates [1,3,11,12,13,14,15,16,17,18,19,20,21,22,23,24,25,26,27,28,29,30,31,32,33,34,35,36,37,38,39,40,41,42,43]. Alarmingly, resistance to ceftazidime-avibactam often emerged during treatment. In these instances, many clinicians switched therapies to meropenem–vaborbactam or imipenem–relebactam. Unlike vaborbactam, which is a monocyclic boronate, relebactam is similar in structure to avibactam. One of the most common resistance mechanisms that KPC-producing Enterobacterales acquire is a single amino acid substitution at Ambler position 179. These variants of KPC possess the characteristics of extended-spectrum β-lactamases (ESBLs) with susceptibility to carbapenems [24,35].

The D179Y amino acid substitution in KPC is one of the most prevalent substitutions found in ceftazidime-avibactam-resistant clinical isolates to date [11,13,14,21,23,35,36,37,39,42,43]. However, the D179N amino acid substitution in KPCs is more problematic as strains that carry this substitution maintain resistance towards penicillins, cephalosporins, aztreonam, narrow-spectrum β-lactam-β-lactamase inhibitor combinations (e.g., ampicillin-clavulanic) and carbapenems [44,45,46]. Unlike the other D179 variants, the D179N variant of KPC-2 is of particular interest because it confers resistance to imipenem, and ceftazidime, in addition to ceftazidime-avibactam; the clinical implications of this “heightened” resistance are alarming [44,47]. The impact of 179 substitutions in KPC on meropenem–vaborbactam was previously evaluated and found to not alter the activity of this combination [7]. Herein, imipenem–relebactam, which is effective at treating Enterobacterales producing KPCs as well as AmpCs and ESBLs, was evaluated against ceftazidime-avibactam resistant KPC-2 variants.

## 2. Results

### 2.1. D179 Variants Are Susceptible to Imipenem–Relebactam When Expressed in Escherichia coli

Susceptibility testing was conducted using imipenem (IMI), imipenem–relebactam (IMI-REL), ceftazidime (CAZ), and ceftazidime-avibactam (CAZ-AVI) using isogenic Escherichia coli strains expressing KPC-2 as well as D179 variants possessing the 19 other amino acid substitutions (Table 1). Only wild-type KPC-2 and the D179N variant tested resistant to imipenem alone (MIC ≥ 4 µg/mL). Notably, the addition of relebactam to imipenem restored susceptibility to all strains. The same strains were highly resistant to ceftazidime and ceftazidime-avibactam with all D179 variants testing resistant to ceftazidime and only one strain (*E. coli* DH10B pBC SK(+) bla_KPC-2_ D179E; MIC = 8 µg/mL) being susceptible to ceftazidime-avibactam.

### 2.2. The D179Y Variant Is Less Catalytically Active Compared to the D179N Variant and KPC-2

**Figure 1:**
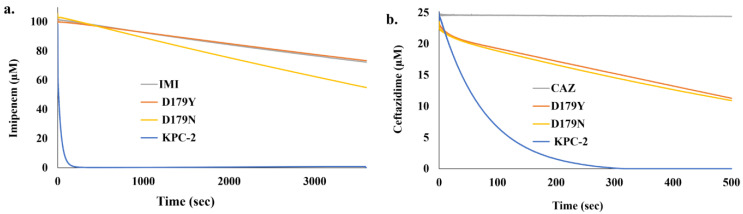
Progress curves for the hydrolysis of (**a**) 100 µM imipenem (IMI) and (**b**) 25 µM ceftazidime (CAZ) by KPC-2 and the D179Y and D179N variants (2 µM enzyme) at 25 °C obtained using a stopped-flow apparatus.

KPC-2 (blue line) and the D179N variant (yellow line) both hydrolyzed imipenem though the overall rate of hydrolysis by the D179N variant was much slower (Figure 1a). The D179Y variant was unable to turnover imipenem (orange line) and was comparable to the background breakdown of imipenem alone (gray line) (Figure 1a). Conversely, all three β-lactamases hydrolyzed ceftazidime. KPC-2 hydrolyzed 25 µM ceftazidime within 300 sec (blue line) (Figure 1b). Conversely, the two variants had slower rates of hydrolysis for ceftazidime, but the rates were similar (yellow and orange lines); the background breakdown of ceftazidime is shown by a gray line (Figure 1b).

The D179N variant possessed a similar *K*_i app_ value for relebactam, as compared to KPC-2, 3.4 ± 0.3 µM and 2.3 ± 0.3 µM, respectively (Table 2). However, the acylation rate of relebactam for the D179N variant was ~2.5x lower than KPC-2. The partition ratios or *k*_inact_/*k*_cat_ values for KPC-2 and the D179N variant were both 1. Due to the poor catalytic turnover by the D179Y variant, inhibition of this variant was not able to be assessed.

### 2.3. Timed Mass Spectrometry Supports the Kinetic Observations That the D179Y Variant Is Slower to Deacylate Imipenem and Ceftazidime, but Also Slower to Be Acylated by Relebactam

**Figure 2:**
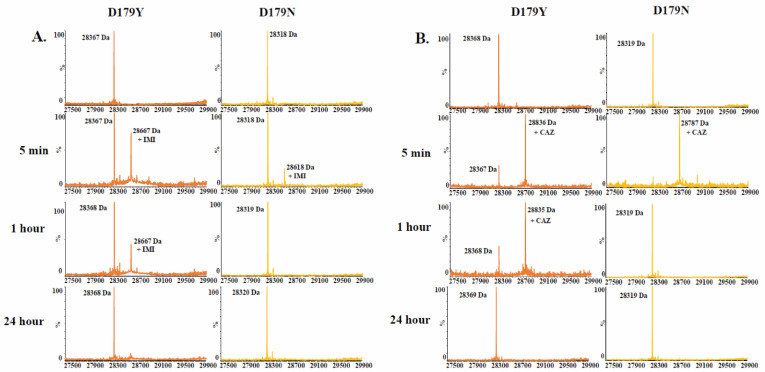
Time-based mass spectrometry (**A**) 1:1 ratio of the D179Y and D179N variants to imipenem. (**B**) 1:50 ratio of variant to ceftazidime.

Time-based mass spectrometry was used to compare the D179N and D179Y variants upon reaction with imipenem, ceftazidime, relebactam, and avibactam. The presence of acyl-complexes with imipenem and ceftazidime with the D179N variant was captured less often compared to the D179Y variant (Figure 2).

**Figure 3:**
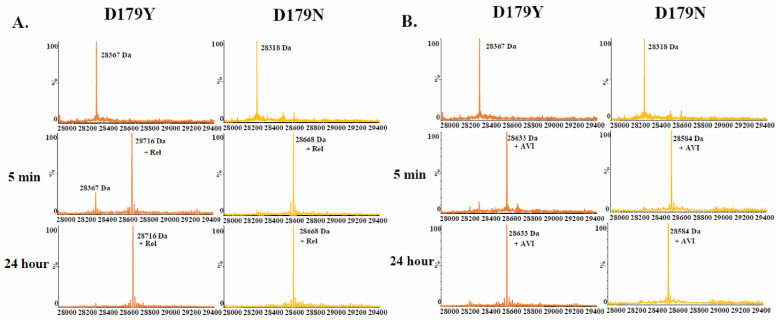
Time-based mass spectrometry (**A**) 1:1 ratio of the D179Y and D179N variants to relebactam. (**B**) 1:1 ratio of variant to avibactam.

Comparing relebactam and avibactam using time-based mass spectrometry revealed that relebactam is slower to form an acyl complex with the D179Y variant compared to avibactam (Figure 3). Relebactam and avibactam behaved similarly vs. the D179N variant. 

### 2.4. Molecular Modeling

**Figure 4:**
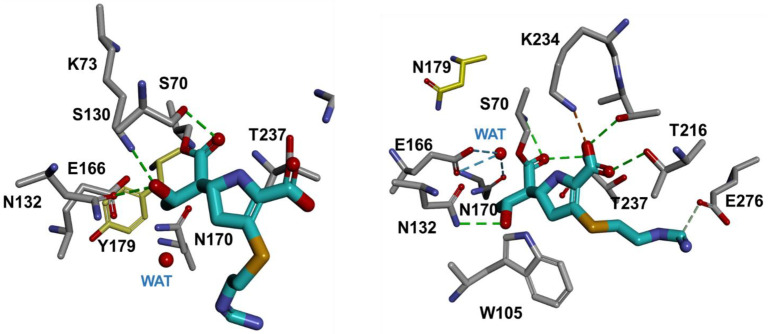
The acyl–enzyme complexes of the D179Y variant (**left**) and the D179N variant (**right**) with imipenem (cyan).

To assess the differences in the biochemical analysis conducted with the purified D179Y and D179N variants, molecular models were constructed with imipenem bound to the nucleophile, S70 when modeling using parameters previously employed [46,49,50]. The D179Y variant revealed that the carbonyl of imipenem is positioned outside of the oxyanion hole, toward S130 (Figure 4a) but inside the oxyanion hole for the D179N variant (Figure 4b). Moreover, the catalytic water is displaced by the ethoxy group of imipenem in the D179Y variant, but not in D179N. 

## 3. Discussion

Imipenem–relebactam was shown to demonstrate antimicrobial activity against ceftazidime-avibactam-resistant D179 variants of KPC-2, including the imipenem-resistant D179N variant. The limitations of our study because of technical challenges, included the susceptibility testing of three *bla*_KPC-2_ mutants in a different vector as well as the inability to measure inhibition kinetics with the D179Y variant. 

Kinetic and time-based mass spectrometry revealed that the D179N variant hydrolyzed imipenem albeit at a slower rate than KPC-2; however, the D179Y variant was unable to turnover imipenem. Both variants hydrolyzed ceftazidime at similar rates, but not as robustly as KPC-2. Despite similar K_i app_ values between KPC-2 and the D179N variant for relebactam, the acylation rate was ~2.5-fold lower for the D179N variant compared to KPC-2. Thus, the D179N variant is not as active as KPC-2 vs. β-lactams and is also less inhibited by relebactam. Time-based mass spectrometry showed that the D179Y variant had an even slower relebactam acylation rate as at 5 min apo-enzyme could still be detected. Thus, the mass spectrometry supported the kinetic observations that the D179Y variant was not as active as the D179N variant. Likely the acylation rate of relebactam for the D179Y variant is even lower than that observed for the D179N variant. Overall, these biochemical parameters translate to imipenem resistance and imipenem–relebactam susceptibility when the D179N variant is expressed in *E. coli*, while the D179Y variant is susceptible to both agents. 

Molecular modeling provided insights into the varying phenotypes of the D179N and D179Y variants vs. imipenem. Upon docking and acylation of imipenem into the active sites of both variants, imipenem is well positioned for hydrolysis in the D179N variant, but not the D179Y variant (i.e., carbonyl positioned outside of the oxyanion hole). Structurally, the D179N variant is more poised for efficient catalysis of imipenem, which supports the increased imipenem hydrolysis by D179N compared to the D179Y variant. The molecular modeling of imipenem is reminiscent of crystal structures of carbapenems bound to class A non-carbapenemases, such as SHV-1 and TEM-1 [51,52]. With SHV-1, the carbonyl of meropenem was found outside and inside the oxyanion hole, while the carbonyl of imipenem was outside the oxyanion hole in TEM-1; both structures support slow hydrolysis of or inhibition by carbapenems. In conclusion, these studies complement previous investigations by elucidating how ceftazidime–avibactam resistant variants at Ambler position 179 (e.g., D179Y) test susceptible to imipenem.

## 4. Materials and Methods

### 4.1. Bacterial Strains and Reagents

The *K. pneumoniae* isolates carrying *bla*_KPC-2_ and pBR322-*catI*-*bla*_KPC-2_ were kind gifts from Fred Tenover of the Centers for Disease Control and Prevention (Atlanta, GA, USA) [53]. Site saturation mutagenesis at the nucleotides corresponding to position 179 in *bla*_KPC-2_ in the pBR322-*catI* plasmid was previously described [44]. Due to technical difficulties, the *bla*_KPC-2 D179E, D179I, or D179S_ genes were purchased from Celtek Genes Service a subdivision of Celtek Bioscience, LLC (Franklin, TN, USA), and cloned into the pBC SK(+) phagemid, as previously described [44]. All plasmids were maintained into *E. coli* DH10B. For protein purification, the construction of pET24a(+)*bla*_KPC-2_ and pET24a(+)bla_KPC-2 D179N_ plasmids was previously described [44,48] and the pET24a(+)*bla*_KPC-2 D179Y_ plasmid was created using a similar approach. These plasmids were maintained into *E. coli* DH10B and transformed into *E. coli* Origami™2 DE3 before protein production.

Imipenem and relebactam were provided by Merck (Merck, Sharp & Dohme, Kenilworth, NJ, USA). Nitrocefin was purchased from Oxoid (ThermoFisher Scientific, Basingstoke, UK). Chloramphenicol was obtained from Sigma-Aldrich (St. Louis, MI, USA). Ceftazidime was procured from Sigma-Aldrich and Research Products International and used interchangeably throughout the experimentation. Avibactam was purchased from Advanced ChemBlocks (cat # R16073). 

### 4.2. Susceptibility Testing

Cation-adjusted Mueller-Hinton (MH) agar-dilution minimum inhibitory concentration (MIC) measurements were performed according to Clinical and Laboratory Standards Institute (CLSI) guidelines [47,54]. A Steers replicator was used to deliver 10-μL of each bacterial inoculum containing 10^4^ CFU per spot directly onto the MH agar plates. Plates were incubated for 16–20 h at 37 °C and MIC values were assessed based on a 100% decrease in growth on the drug plates compared to the MH-only control plate. Relebactam and avibactam were tested at a constant 4 µg/mL. MIC measurements were performed in at least triplicate. 

### 4.3. Protein Expression and Purification

The KPC-2, D179N, and D179Y variant β-lactamases were purified from *E. coli* Origami™2 DE3 (Novagen, Burlington, ON, USA), as previously described [44,48]. Briefly, cells were grown in super optimal broth (SOB) at 37 °C to an optical density at λ_600 nm_ (OD600) of approximately 0.6–0.8 and induced with 0.5 mM isopropyl β-d-1-thiogalactopyranoside (IPTG) for a minimum of three hours to express the β-lactamase. Pelleted cells were frozen and lysed with lysozyme and the supernatants were further purified by preparative isoelectric focusing and fast protein liquid chromatography (FPLC) using a HiTrap Q anion-exchange chromatography column. Proteins were frozen with 25% glycerol and stored at −20 °C. The purity of the proteins was assessed by mass spectrometry. Protein concentrations were determined by measuring absorbance at a wavelength of λ_280 nm_ and using the protein’s extinction coefficient (Δε 39,545 M^−1^ cm^−1^) obtained using the ProtParam tool at ExPASy Bioinformatics Resource Portal [55].

### 4.4. Kinetics

For progress curves of imipenem and ceftazidime hydrolysis, 2.0 μM of KPC-2 and the D179Y and D179N variants were incubated with either 100 μM imipenem or 25 μM ceftazidime in sterile 10 mM PBS pH 7.4 at 25 °C. Data were collected using an Applied Photophysics SX20 Stopped Flow spectrophotometer (260 nm) with the ProData SX software version 1.0.

Kinetic inhibition assays were conducted using an Agilent 8453 diode array spectrophotometer in 10 mM phosphate-buffered saline, pH 7.4 at room temperature. Determination of the values of the kinetic constants apparent K_i_ (K_i app_), k_2_/K, and k_cat_/k_inact_, was previously described [56].

### 4.5. Timed Mass Spectrometry

The D179Y and D179N variants were incubated with imipenem, relebactam, or avibactam at an enzyme/compound molar ratio of 1:1 and ceftazidime at a molar ratio of 1:50 in sterile 10 mM phosphate-buffered saline (PBS) at pH 7.4 for 5 min and 24 h. Reactions were stopped by adding 10 μL acetonitrile and samples were transferred into 0.1% formic acid in water. Samples were analyzed using Q-TOF Waters Synapt-G2-Si and Waters Acquity UPLC BEH C18 1.7 µm column (2.1 mm × 50 mm), as previously described [44]. 

### 4.6. Molecular Modeling

The crystal coordinates of KPC-2 (PDB:2OV5) were used to generate structural representations of the D179Y and D179N variants using Discovery Studio (Acclerys Inc., San Diego, CA, USA) as previously described [49,50,57]. The crystallographic water molecules were maintained during modeling. The KPC-2 β-lactamase structure and the variant model were solvated and minimized to an RMS of 0.03 Å using the Conjugate Gradient method. Acyl–imipenem was constructed using the Fragment Builder tools and minimized using a Standard Dynamics Cascade protocol of Discovery Studio. The acylated imipenem was automatically docked into the active site of the D179Y and D179N variants using the CDOCKER module of Discovery Studio. 

## 5. Conclusions

These studies also complement previous investigations [44,45,46,47] by proposing hypotheses to explain why variants such as KPC-2 D179Y became susceptible to imipenem when expressed in *E. coli*. Our data suggest that imipenem adopts a catalytically unfavorable conformation in the active site reminiscent of studies done with other carbapenems in non-carbapenemase β-lactamases [51,52]. Thus, imipenem serves as a “β-lactamase inhibitor.” Importantly, there also seems to be a reduction in the ability of relebactam to inactivate D179Y compared to avibactam, although this is not evident by susceptibility testing. The implication of these paradoxical findings only further compels us to investigate these complex β-lactamases to anticipate future development of resistance as new combinations are introduced into the clinic. Moreover, novel KPC variants that confer ceftazidime–avibactam resistance will pose even more challenges as they become more prevalent [58].

## Figures and Tables

**Table 1:** Susceptibility testing results for Ambler position D179 site-saturation *bla*_KPC-2_ mutants expressed in *E. coli* DH10B.

Strain	IMI	IMI- REL	CAZ	CAZ- AVI
*Klebsiella pneumoniae bla*_KPC-2_	8	1	64	1
*E. coli* DH10B	0.5	0.25	0.5	0.25
*E. coli* DH10B pBC SK(+)	0.25	0.25	0.5	0.25
*E. coli* DH10B pBC SK(+) *bla*_KPC-2_	0.5	0.25	1	0.5
*E. coli* DH10B pBC SK(+) *bla*_KPC-2_ D179E	0.5	0.5	16	8
*E. coli* DH10B pBC SK(+) *bla*_KPC-2_ D179I	0.5	0.25	256	16
*E. coli* DH10B pBC SK(+) *bla*_KPC-2_ D179S	0.5	0.5	256	32
*E. coli* DH10B pBR322 *bla*_KPC-2_	8	0.5	64	1
*E. coli* DH10B pBR322 *bla*_KPC-2_ D179A	0.5	0.5	512	32
*E. coli* DH10B pBR322 *bla*_KPC-2_ D179R	0.5	0.5	16	16
*E. coli* DH10B pBR322 *bla*_KPC-2_ D179N	4	0.5	512	16
*E. coli* DH10B pBR322 *bla*_KPC-2_ D179C	1	0.5	256	32
*E. coli* DH10B pBR322 *bla*_KPC-2_ D179Q	0.5	0.5	128	32
*E. coli* DH10B pBR322 *bla*_KPC-2_ D179G	1	0.5	512	32
*E. coli* DH10B pBR322 *bla*_KPC-2_ D179H	0.5	0.5	256	32
*E. coli* DH10B pBR322 *bla*_KPC-2_ D179L	0.5	0.5	512	64
*E. coli* DH10B pBR322 *bla*_KPC-2_ D179K	0.5	0.5	32	16
*E. coli* DH10B pBR322 *bla*_KPC-2_ D179M	0.5	0.5	512	64
*E. coli* DH10B pBR322 *bla*_KPC-2_ D179F	0.5	0.5	512	64
*E. coli* DH10B pBR322 *bla*_KPC-2_ D179P	0.5	0.5	256	64
*E. coli* DH10B pBR322 *bla*_KPC-2_ D179T	0.5	0.5	256	64
*E. coli* DH10B pBR322 *bla*_KPC-2_ D179W	0.5	0.5	>512	32
*E. coli* DH10B pBR322 *bla*_KPC-2_ D179Y	0.5	0.5	512	64
*E. coli* DH10B pBR322 *bla*_KPC-2_ D179V	0.5	0.5	512	64

Abbreviations: imipenem (IMI), relebactam (REL), ceftazidime (CAZ), and avibactam (AVI). Relebactam and avibactam were maintained at 4 µg/mL. The Clinical Laboratory Standards Institute’s (CLSI) breakpoints (S, susceptible (green); I, intermediate; R, resistant (red)) for the tested compounds are as follows: imipenem: S ≤ 1; I = 2; R ≥ 4; imipenem–relebactam: S ≤ 1/4; I = 2/4; R ≥ 4/4; ceftazidime: S ≤ 4; I = 8; R ≥ 16; and ceftazidime-avibactam: S ≤ 8/4; R ≥ 16/4. The majority (16/19) of the D179 variants were generated using the pBR322-*catI*-*bla*_KPC-2_ plasmid; however, due to technical issues, the remainder, D179E, -I, and -S variants were constructed from the pBC SK(+) *bla*_KPC-2_ plasmid. The basal expression level of *bla*_KPC-2_ was previously shown to be higher from the pBR322-*catI*-*bla*_KPC-2_ plasmid than from the pBC SK(+) *bla*_KPC-2_ plasmid; thus, those strains have higher overall MICs [48].

**Table 2:** Kinetic inhibition constants of the D179N variant compared to wild-type KPC-2.

Relebactam	*K* _i app_ (µM)	*k*_2_/*K* (M^−1^s^−1^)	*k*_cat_/*k*_inact_
KPC-2	2.3 ± 0.3 ^#^	24,750 ± 2475 ^#^	1 ^#^
D179N	3.4 ± 0.3	9975 ± 998	1

# Previously reported in [6].

## Data Availability

Data is available upon request.
